# Strategies to improve treatment coverage in community-based public health programs: A systematic review of the literature

**DOI:** 10.1371/journal.pntd.0006211

**Published:** 2018-02-08

**Authors:** Katrina V. Deardorff, Arianna Rubin Means, Kristjana H. Ásbjörnsdóttir, Judd Walson

**Affiliations:** 1 Department of Global Health, University of Washington, Seattle, Washington, United States of America; 2 DeWorm3, Natural History Museum, London, United Kingdom; 3 Departments of Medicine, Pediatrics, and Epidemiology, University of Washington, Seattle, Washington, United States of America; London School of Hygiene and Tropical Medicine, UNITED KINGDOM

## Abstract

**Background:**

Community-based public health campaigns, such as those used in mass deworming, vitamin A supplementation and child immunization programs, provide key healthcare interventions to targeted populations at scale. However, these programs often fall short of established coverage targets. The purpose of this systematic review was to evaluate the impact of strategies used to increase treatment coverage in community-based public health campaigns.

**Methodology/ principal findings:**

We systematically searched CAB Direct, Embase, and PubMed archives for studies utilizing specific interventions to increase coverage of community-based distribution of drugs, vaccines, or other public health services. We identified 5,637 articles, from which 79 full texts were evaluated according to pre-defined inclusion and exclusion criteria. Twenty-eight articles met inclusion criteria and data were abstracted regarding strategy-specific changes in coverage from these sources. Strategies used to increase coverage included community-directed treatment (n = 6, pooled percent change in coverage: +26.2%), distributor incentives (n = 2, +25.3%), distribution along kinship networks (n = 1, +24.5%), intensified information, education, and communication activities (n = 8, +21.6%), fixed-point delivery (n = 1, +21.4%), door-to-door delivery (n = 1, +14.0%), integrated service distribution (n = 9, +12.7%), conversion from school- to community-based delivery (n = 3, +11.9%), and management by a non-governmental organization (n = 1, +5.8%).

**Conclusions/significance:**

Strategies that target improving community member ownership of distribution appear to have a large impact on increasing treatment coverage. However, all strategies used to increase coverage successfully did so. These results may be useful to National Ministries, programs, and implementing partners in optimizing treatment coverage in community-based public health programs.

## Author summary

Many public health platforms provide decentralized interventions outside of health facilities, including mass drug administration for neglected tropical diseases, immunizations, vitamin supplementation, and others. The purpose of these community-based public health platforms is to reach large proportions of populations in need with important preventative healthcare. However the platforms require high treatment coverage of targeted populations in order to achieve health impact. And, in many cases, targeted populations are low-income, rural, and hard to reach with large health campaigns. The purpose of this systematic review is to evaluate strategies for achieving high treatment coverage in public health service distribution programs. We identified nine different strategies used to increase coverage of distribution programs. Community-directed distribution was associated with the largest increase in treatment coverage. Similarly, incentivizing distributors also had a strong influence on increasing treatment coverage. These findings have important implications for governments, implementers, and funders who aim to provide health services at scale.

## Introduction

The health impact of community-based public health programs is dependent upon the proportion of the targeted population that is reached with the preventative health services, also known as treatment coverage. The World Health Organization (WHO), country governments, and other institutions typically establish specific treatment coverage targets for community-based programs in order to benchmark programmatic success and achieve specific health outcomes. For example, the WHO recommends mass drug administration (MDA) treatment coverage with praziquantel and albendazole of at least 75% of children aged 6–15 years in schistosomiasis and soil transmitted helminth (STH) endemic areas. However, community-based public health programs often fail to achieve the pre-established coverage targets required to reduce morbidity, interrupt the transmission of diseases, or establish herd immunity [1].

Achieving high treatment coverage in the delivery of health services is critical to the success of community-based public health programs such as vitamin A supplementation (VAS), child immunizations, and MDA campaigns targeting neglected tropical diseases (NTDs). In order to reach large proportions of a population, these programs require strong public health platforms, typically decentralized outside of healthcare facilities. Delivery mechanisms include distribution through schools with or without the involvement of health professionals, delivery at community gathering points, or door-to-door distribution by health staff or volunteer community drug distributors (CDDs).

Challenges to achieving high treatment coverage include insufficient and inappropriate delivery systems, geographically remote populations, urban populations with high migration, competing resource and time priorities of distributors, and community misunderstanding or mistrust of the program [2–6]. For example, use of school-based delivery platforms to target school-aged children in areas with low school attendance rates compromises treatment coverage [7]. In Mali, Nigeria, and Sierra Leone, an insufficient number of volunteer drug distributors and low motivation among distributors was correlated with low MDA treatment coverage in lymphatic filariasis (LF) programs [2]. Additionally, community skepticism of LF MDA programs was associated with low participation of adults and adolescents in Tanzania. Mistrust was the result of community misunderstanding of the reason for distribution, a lack of information sharing, and fear of adverse side effects [3].

Given the multiple challenges to achieving high treatment coverage in community-based programs, it is important to identify and evaluate specific strategies that may have utility in increasing or sustaining high coverage. Sustaining high coverage is not only important for achieving health impact at the population level, but is also necessary within the context of a rights-based approach to health in order to ensure that individuals or groups are not repeatedly excluded from important health services. With this goal in mind, we sought to identify strategies that have been used to increase coverage in community-based programs and to evaluate their influence on treatment coverage. In doing so, we aim to generate evidence that may be useful to scaling community-based public health programs more broadly.

## Materials and methods

### Search strategy and selection criteria

This systematic review was conducted in accordance with the Preferred Reporting Items for Systematic Review and Meta-Analysis (PRISMA) guidelines [8]. We systematically searched the electronic databases CAB Direct, Embase, and PubMed for articles that reported two or more treatment coverage estimates from community-based distribution of drugs, vaccines, or other public health services prior to and following an intervention intended to increase coverage. We used the search string (“child health days*” OR chemoprevention OR “community based treatment” OR “community-directed” OR “community engagement” OR “deworm*” OR “mass drug administration” OR “preventative chemotherapy” OR “public health program” OR “supplementary immunization” OR “vaccin* campaign” OR “vitamin A”) AND (coverage OR compliance OR adherence) AND “humans.” No restrictions were made on study location, date of publication, article type, or language.

Titles and abstracts were screened for all articles. All abstracts that described treatment coverage of a vaccine campaign, MDA, or other community-based distribution were included for full text review. If it was unclear if a study met inclusion criteria based upon the title and abstract, the text was read in full. According to pre-defined inclusion criteria, articles were excluded if they focused on facility-based distribution rather than community-based distribution. Articles that described supplemental or catch up campaigns were also excluded, as these represent a programmatic reaction to low coverage rather than a strategic intervention to improve coverage. The primary outcome of interest in this review was the measurable difference in treatment coverage of community-based public health distribution prior to and following implementation of interventions aimed at increasing coverage. Therefore, articles that qualitatively described barriers and/or methods to obtaining high coverage without reporting coverage estimates were excluded. Authors of included articles were contacted to clarify the study design or results, if needed.

Article review and data abstraction were performed by a reviewer (KVD) and, in any circumstances where the eligibility of an article was unclear, two additional reviewers were consulted (ARM and KHA).

### Data abstraction

A structured data abstraction tool was used to abstract data from articles reviewed in full. Data abstracted from full texts included details on the intervention used to increase coverage, the size of the targeted and treated populations, dates of distribution, coverage estimates for baseline or control populations (pre-intervention), and coverage estimates for follow up or experimental populations (post intervention). Study design, data source, location, urban or rural setting of the distribution, and targeted disease were also collected.

### Meta-analysis

Individual treatment coverage estimates reported in articles were confirmed using reported number of targeted and treated individuals. If the 95% confidence interval (CI) for the coverage estimate was not reported, it was calculated using a one-sample proportion test and the sample size reported in the article. If the number of targeted or treated individuals was not reported in the article, it was calculated based upon the sample size and coverage estimates reported. If no sample size was reported, the corresponding author was contacted and requested to provide further details. Pooled coverage estimates for each strategy category were calculated by combining the sample sizes reported in each relevant study and using a one-sample proportion test to calculate proportion covered with 95% CIs. Percent change in treatment coverage for each study was calculated as the difference of pre- and post-intervention coverage estimates. All data analysis was conducted using the statistical computing software R (R Foundation for Statistical Computing, Vienna, Austria) and forest plots produced using the “forest plot” package [9, 10].

## Results

**Fig 1:**
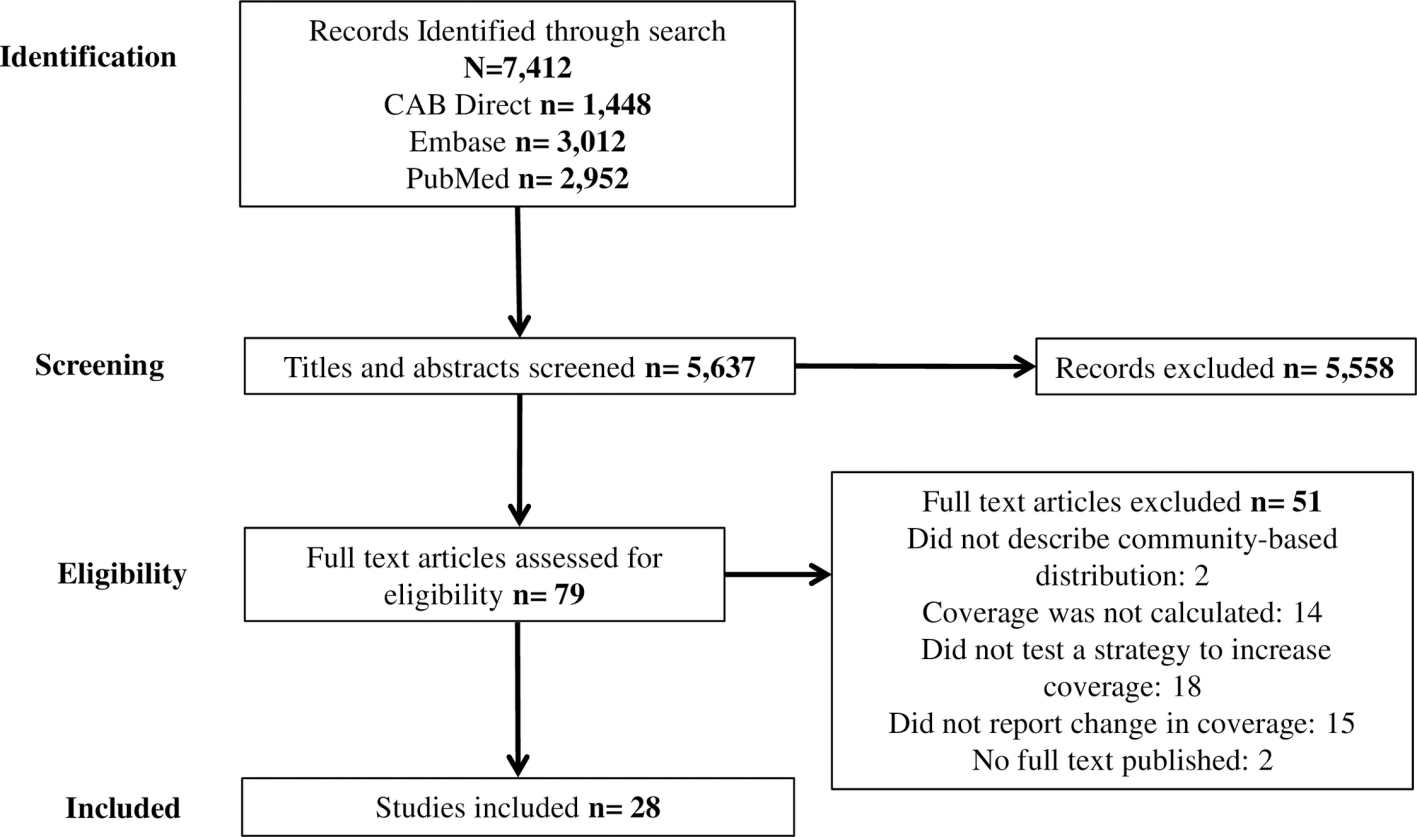
Inclusion diagram of studies reviewed in accordance with Preferred Reporting Items for Systematic Reviews and Meta-Analyses (PRISMA).

We conducted the database search on October 2, 2017. We identified 5,637 unique studies from which 79 full text articles were evaluated for possible inclusion (Fig 1). Of those, two articles were excluded because they did not describe community-based distribution, fourteen were excluded because they did not report coverage estimates, eighteen were excluded because they did not describe a strategy to increase coverage, fifteen reported only a single coverage estimate, and two articles had not yet been published as full texts at the time of the review.

After full text review, 28 articles describing 9 strategies to increase coverage met inclusion criteria. Strategies included community-directed treatment distribution (n = 6) [4, 7, 11–14], distribution within kinship networks (n = 1) [15], distributor incentives (n = 2) [16, 17], intensified information, education, and communication (IEC) activities (n = 8) [5, 18–24], conversion from door-to-door to fixed-point delivery (n = 1) [20], conversion from fixed-point to door-to-door delivery (n = 1) [25], integrated service distribution (n = 9) [19, 26–33], conversion from school- to community-based delivery (n = 3) [7, 32, 34], and management by a non-governmental organization (NGO) rather than ministry of health (MOH) (n = 1) [35]. Four of the studies used combinations of these strategies. One study reported a combination incorporating both community-based distribution and community-directed treatment strategies [7], one study incorporated both community-based distribution and integration [32], one study incorporated both integrated service distribution and intensified IEC [19], and one study reported combining intensified IEC activities and fixed-point distribution [20].

Studies used a variety of methodological approaches to evaluate the change in coverage of several public health services including child health services, preventative chemotherapy for NTDs, and delivery of various vaccines (Tables 1 and 2). Distribution activities took place in 28 countries in Africa, Asia, South America, Eastern Europe, and the Middle East (Table 2).

**Table 1:** Study design characteristics of included articles.

First Author	Study Design or Analysis	Baseline or Control	Strategy Implemented
Akogun 2012	Prospective cross-sectional measurements in control and experimental areas	No distribution program at baseline or in control	Community-directed distribution
Babu 2006	Simultaneous community-RCT in control and experimental areas	Standard community-based MDA activity (control)	Community-directed MDA
Blackburn 2006	Prospective cross-sectional measures in one area	No distribution program (baseline)	Integrated distribution with existing MDA
Calderon-Ortiz 1996	Simultaneous community-RCT in control and experimental areas	Standard periodic vaccine campaigns	Permanent hire of community vaccine distributors
Cantey 2010	Simultaneous community-RCT in control and experimental areas	Standard community-based MDA activity (control)	Intensified education campaign for community-based MDA
Dembele 2012	Prospective cross-sectional measures in one area	Vertical distribution programs	Integrated distribution
Doherty 2010	Retrospective cross-sectional measures from DHS data	Distribution of child health services pre-CHD implementation (baseline)	Integrated distribution of child health services through CHDs
Goodson 2012	Cross-sectional measures in two areas	Integrated distribution not including ITN	Integrated distribution including ITN
Grabowsky 2005	Prospective cross-sectional measures in one area	No distribution program (baseline)	Integrated distribution with existing vaccine campaign
Gyapong 2000	Simultaneous community-RCT in control and experimental areas	Standard health services-directed MDA	Community-directed MDA
Habib 2017	Simultaneous community-RCT in control and experimental areas	Standard vertical distribution	Enhanced IEC and service integration
Halwindi 2010	Simultaneous community-RCT in control and experimental areas	Health services-directed distribution through Child Health Weeks	Community-directed distribution
Katabarwa 2010	Simultaneous community-RCT in control and experimental areas	Standard community-directed MDA	Kinship-enhanced community-directed MDA
King 2011	Prospective cross-sectional measures in one area	Standard school-based MDA (baseline)	Fixed-point community distribution with intensified awareness campaign
Ladner 2014	Linear regression of retrospective Gaurdasil Access Program (GAP) data	—	Management source
Linkins 1995	Prospective cross-sectional measures in one area	Fixed-point community distribution (baseline)	Door-to-door MDA distribution
Massa 2009	Simultaneous community-RCT in control and experimental areas	Standard school-based MDA (control)	Community-based MDA
Muhumuza 2013	Prospective cross-sectional measures in one area	Standard school-based MDA (baseline)	Increased teacher motivation with training and incentives for school-based MDA
Mwingira 2016	Prospective cross-sectional measures in same area	Vertical distribution (baseline)	Integrated distribution
Ndyomugyenyi 2003	Simultaneous community-RCT in two control and one experimental area	School-based distribution, and standard community-directed distribution	community-based community-directed distribution, and integrated community-directed distribution
Njomo 2014	Simultaneous community-RCT in control and experimental areas	Standard community-based MDA activity (control)	Intensified awareness campaign for community-based MDA
Oliphant 2010	Retrospective cross-sectional measures from DHS data	Distribution of child health services pre-CHD implementation (baseline)	Integrated distribution of child health services through CHDs
Oshish 2011	Prospective cross-sectional measures in one area	Standard school-based MDA (baseline)	Community-directed community-based MDA
Rahman 2012	Prospective cross-sectional measures in one area	Standard community-based vaccine delivery (baseline)	Education campaign with support of local religious leaders
Ramaiah 2006	Prospective cross-sectional measures in one area	Standard community-based MDA (baseline)	Intensified awareness campaign for community-based MDA
de Rochars 2005	Prospective cross-sectional measures in one area	Standard community-based MDA (baseline)	Intensified awareness campaign for community-based MDA
Wamae 2006	Simultaneous community-RCT in control and experimental areas	Standard health services-directed MDA	Community-directed MDA
Zimicki 1994	Prospective cross-sectional measures in one area	Standard routine vaccine distribution	Mass media communication campaign

**Table 2:** Disease focus, location, and setting of included articles.

Disease Focus	First Author	Location	Setting
Child Health Services (ex. Vitamin A)	Doherty 2010	Madagascar, Tanzania, Zambia, Zimbabwe	National
	Goodson 2012	Madagascar	National
	Halwindi 2010	Zambia	Rural
	Oliphant 2010	Ethiopia, Madagascar, Uganda, Tanzania, Zambia, Zimbabwe	National
Childhood Vaccines	Calderon-Ortiz 1996	Mexico	Urban
	Rahman 2012	Iraq	Mixed
	Zimicki 1994	Philippines	National
HPV Vaccine	Ladner 2014	Bhutan, Bolivia, Cambodia, Cameroon, Georgia, Haiti, Honduras, Kenya, Lesotho, Moldova, Nepal, Tanzania, Uganda, Uzbekistan	Mixed
ITN	Akogun 2012	Nigeria	Rural
	Blackburn 2006	Nigeria	Rural
	Grabowsky 2005	Zambia	Mixed
Lymphatic Filariasis	Babu 2006	India	Urban
	Cantey 2010	India	Rural
	Gyapong 2000	Ghana	Rural
	King 2011	American Samoa	Mixed
	Njomo 2014	Kenya	Urban
	Ramaiah 2006	India	Mixed
	de Rochars 2005	Haiti	Mixed
	Wamae 2006	Kenya	Rural
Onchocerciasis	Katabarwa 2010	Uganda	Rural
OPV	Habib 2017	Pakistan	Mixed
	Linkins 1995	Egypt	Mixed
Schistosomiasis	Massa 2009	Tanzania	Rural
	Muhumuza 2013	Uganda	Mixed
	Oshish 2011	Yemen	Mixed
Integrated NTD and PH Services	Dembele 2012	Mali	National
	Mwingira 2016	Tanzania	National
	Ndyomugyenyi 2003	Uganda	Mixed

HPV: Human Papiloma Virus; ITN: Insecticide treated net; OPV: Oral Polio Vaccine; STH: Soil Transmitted Helminthes; PH: Public Health

**Fig 2:**
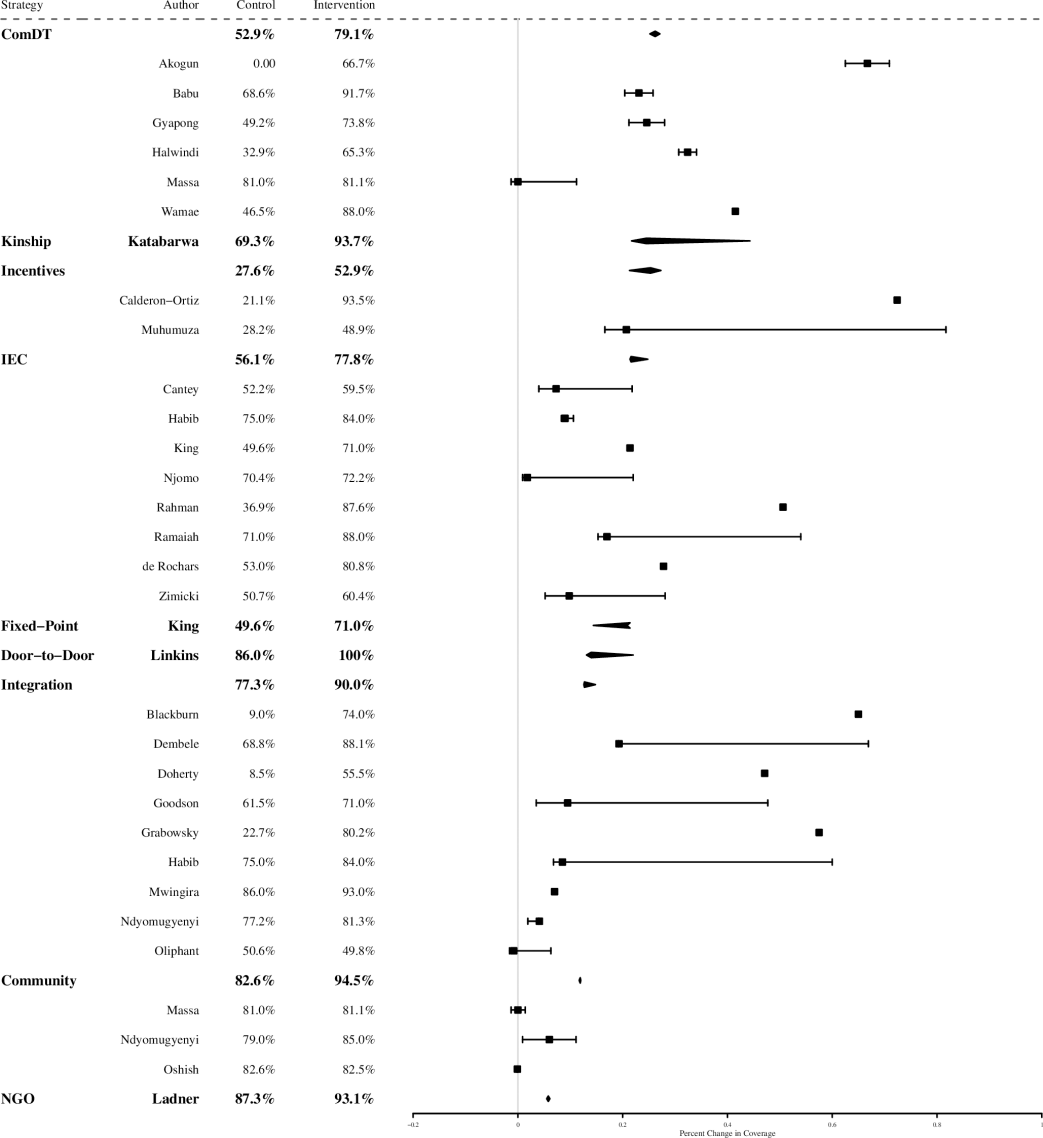
Percent change in coverage achieved by each strategy and individual study. (IEC: information, education, and communication activities; Community: community-based delivery; ComDT: community-directed treatment).

Fig 2 summarizes the percent change in coverage achieved by each strategy category and individual intervention, described in the following sections. Strategies that demonstrated the largest positive influence on treatment coverage in the 28 included studies were: 1) community-directed distribution (6 studies, pooled percent change in coverage = +26.2%), 2) incentives to increase distributor motivation (2 studies, +25.3%), 3) distribution along kinship networks (1 study, +24.5%), and 4) implementation of intensified IEC activities preceding service distribution (8 studies, +21.6%). Strategies that demonstrated the highest absolute post-intervention coverage were: 1) door-to-door distribution (1 study, pooled coverage = 100%), 2) community-based delivery (3 studies, 94.5%), and 3) distribution along kinship networks (1 study, 93.7%). There did not appear to be any trend in the success of strategies by disease or location.

### Community-directed treatment vs. standard-of-care delivery

Six studies described MDA campaigns that were designed and delivered via a strategy of community-directed treatment (ComDT) compared to standard-of-care delivery [4, 7, 11–14]. The ComDT approach to delivery is characterized by efforts to systematically solicit community input in the organization and implementation of service delivery [36]. We have summarized these six studies briefly below.

Nomadic communities in Nigeria are a hard-to-reach community for public health services. Akogun 2012 evaluated whether the ComDT approach could increase household ownership of insecticide-treated nets (ITN) compared to standard ITN distribution using a prospective study of experimental and control communities [11]. The experimental communities used ComDT to develop culturally acceptable methods for collecting and distributing ITNs, while the control communities continued to rely on standard distribution of ITNs. Coverage was defined as the percentage of households that owned an ITN. Each community had a baseline ITN coverage of zero. Comparing prospective cross sectional measures of ITN coverage after twelve months, Akogun 2012 found that ComDT distribution increased ITN coverage to 66.7% in the experimental community, while ITN coverage remained at zero in the control community (Fig 2).

A community randomized controlled trial (RCT) in an urban setting in Orissa, India (Babu 2006) evaluated the influence of ComDT delivery on treatment coverage in an LF MDA program [4]. Community participation in this study included establishing a steering committee of representatives from the local primary health center, local community-based organizations (CBOs), NGOs, journalists, and government. The steering committee made key decisions concerning dates and locations of distribution, identifying community partners, selecting and training community drug distributors, and designing the accompanying social mobilization campaign. Standard-of-care MDA activities that did not systematically elicit community involvement were implemented in the control clusters. Coverage was calculated as the proportion of eligible community members who received treatment. ComDT delivery achieved 91.7% coverage compared to 68.6% coverage in control clusters (Fig 2).

Gyapong 2000 used a community-RCT in four districts in Ghana to evaluate the influence of ComDT distribution on LF treatment coverage compared to standard MDA distribution [12]. In experimental communities, local health facility staff collaborated with community leaders to organize the MDA distribution method prior to drug distribution. Coverage was defined as the percent population who received treatment. Standard MDA was conducted in control communities. ComDT distribution achieved 73.8% treatment coverage, while standard MDA achieved 49.2% treatment coverage (Fig 2).

The standard method for distribution of child health services in the Southern Province of Zambia is through government-run Child Health Weeks. Halwindi 2010 used a community-RCT to evaluate how ComDT could increase Child Health Week coverage [13]. Standard-of-care Child Health Week services were provided at health facilities and outreach posts in control sites. In experimental sites, services were distributed both at health facilities, as well as by community drug distributors through door-to-door and fixed-point distribution, as designed by the community. Coverage was defined as the percent of children aged 12 to 59 months who received services. Average treatment coverage achieved over four standard Child Health Weeks was 32.9%, while average treatment coverage achieved over four ComDT Child Health Weeks was 65.3% (Fig 2).

A community-RCT in Tanzania compared the effect of a community-based ComDT approach to schistosomiasis and STH MDA delivery to standard-of-care school-based delivery (Massa 2009) [7]. Villages selected their own community drug distributors, the drug delivery platform, and the timing of the campaign. Treatment coverage from two rounds of distribution was evaluated. The average change in treatment coverage from school-based to ComDT delivery among enrolled children was -1.5%, but 23.6% among unenrolled children (Fig 2).

Wamae 2006 used a community-RCT in Kenya to compare the influence of ComDT on coverage of LF MDA as compared to standard MDA delivery [14]. Interactive trainings were conducted with health staff, community leaders, and community drug distributor volunteers on LF biology and the ComDT approach before drug distribution. Treatment coverage was defined as the percentage of people who received and swallowed tablets in each study arm. While standard LF MDA achieved 46.5% treatment coverage, ComDT achieved 88.0% treatment coverage (Fig 2).

The six studies demonstrated a pooled increase in coverage of 26.2%, and an average absolute post-intervention coverage of 79.1% (Fig 2).

### Kinship-enhanced community-directed treatment vs. standard community-directed treatment

Building upon the concept of ComDT, Katabarwa 2010 evaluated if MDA treatment coverage is higher in areas where people tend to live near relatives due to the leveraging of kinship networks [15]. Kinship-networks utilize the same approach as ComDT distribution, however the distribution is conducted within kinship groups. This approach requires more drug distributors than the standard approach as distribution is targeted to smaller groups. Coverage was defined as the percent population who received treatment for onchocerciasis. While ComDT alone achieved an average treatment coverage of 69.3% over two years, kinship-enhanced distribution achieved an average treatment coverage of 93.7% over that same period (Fig 2).

### Distributor incentives vs. no incentives

Calderon-Ortiz 1996 used a community-RCT in Mexico to evaluate the influence of permanently hiring community vaccine distributors on the percentage of children less than one year of age who complete their vaccine schedules [17]. This was contrasted with standard vaccine campaigns distributed by temporary vaccine distributors. Coverage was defined as the percent of children less than one year in age who completed their vaccine schedule. While the standard vaccine campaign resulted in 21.1% coverage, permanently hiring community distributors to provide vaccines resulted in 93.5% coverage (Fig 2).

Muhmuza 2013 used prospective repeat cross-sectional measures to evaluate the influence of drug distributor incentives on schistosomiasis MDA treatment coverage in 12 primary schools in Uganda [16]. Incentives for the teachers and health staff that distributed drugs consisted of a one-day refresher course on drug distribution and t-shirts printed with “NTD Control Program.” Coverage was calculated as the proportion of targeted children who received treatment. Standard-of-care distribution without distributor incentives resulted in coverage of 28.2% of children while the incentives strategy implemented the following year resulted in coverage of 48.9% (Fig 2).

The pooled increase in treatment coverage from these two studies was 25.3%, with an average absolute post-intervention coverage of 52.9% (Fig 2).

### Enhanced information, education, and communication activities vs. standard activities

Eight studies used intensified IEC activities, including the development and distribution of posters, flyers, leaflets, brochures, and radio and TV broadcasting of health messaging in the local language to promote awareness of distribution activities, provide health education, and promote behavior change, to increase treatment coverage [5, 18–24, 37]. Coverage of each MDA program was calculated as the proportion of the total targeted population that received treatment.

A community-RCT in Orissa State, India evaluated the influence of intensified IEC activities on LF MDA treatment coverage when implemented in the month preceding MDA (Cantey 2010) [18]. Compared to the standard pre-MDA education campaign promoted by MOH, IEC activities in experimental clusters consisted of murals, street plays, radio and newspaper advertisements, village education sessions, and increased dissemination of posters and leaflets. The IEC materials described LF transmission mechanisms, drug side effects, and mosquito control measures. Experimental clusters achieved 59.5% MDA treatment coverage compared to 52.2% in control clusters (Fig 2).

Habib 2017 used a community-RCT to evaluate the influence of an IEC campaign and integrated distribution on OPV coverage in Pakistan [19]. The control arm used routine vertical immunization services, while the experimental arm used integrated distribution of OPV and maternal and child health services preceded by IEC activities. IEC materials included information on maternal health services, nutrition, hygiene and sanitation, and OPV. Community mobilizers distributed IEC materials with the aid of pictorial booklets and counseling cards for reference. Coverage was defined as the percent of children aged 1 month to five years who received OPV. In the control arm, where OPV was distributed vertically with no accompanying IEC, OPV treatment coverage was 75%. In the experimental arm, where OPV was integrated with other services and incorporated IEC materials, OPV treatment coverage was 82% (Fig 2).

A study in American Samoa used prospective repeat cross-sectional sampling to evaluate IEC-associated changes in LF MDA coverage (King 2011) [20]. The intensified behavior change campaign consisted of brief radio and television broadcasts of skits, testimonials, and announcements describing LF transmission and prevention, and locations of MDA distribution. Broadcasts were made during morning shows, before local news broadcasts, and during high profile sporting events prior to and during MDA. Treatment coverage increased from 49.6% at baseline to 71.0% at follow up (Fig 2). The study also transitioned MDA from door-to-door delivery method to fixed-point delivery, as described in the following section.

A community-RCT in urban Kenya evaluated the effect of an enhanced IEC campaign on LF MDA treatment coverage in an area with historically low campaign participation (Njomo 2014) [5]. In experimental clusters, MDA was preceded by increased dissemination of posters and banners in public places, announcements made by loudspeaker on the day before and day of distribution, and increased announcements made from mosques, churches, and schools. Experimental clusters that implemented the enhanced IEC campaign achieved 72.2% coverage, compared to 70.4% in control clusters conducting standard IEC activities (Fig 2).

In Akre District, Iraq, an area with historically low childhood vaccine coverage, Rahman 2012 used a prospective cross-sectional study to evaluate how implementation of an IEC campaign that included messaging from community religious leaders influenced vaccine coverage [21]. Coverage was defined as the percent of children less than one year in age who received vaccines. Standard vaccine distribution at baseline was conducted with no accompanying education program one to three times per week at health clinics or by mobile outreach teams, achieving 36.9% coverage of children less than one year of age. When the research team implemented an IEC program preceding vaccine distribution, including announcements over loudspeakers, health talks, posters, and video, vaccine coverage increased to 87.6% (Fig 2).

A prospective repeat cross-sectional study in India compared LF MDA treatment coverage after an enhanced communication campaign to standard-of-care (Ramaiah 2006) [22]. The communication campaign consisted of increased distribution of registration slips, posters, and badges compared to the standard-of-care. The campaign also introduced novel IEC activities such as distribution of ribbon flags, leaflets, t-shirts, cotton bags, and malted drink sachets, in addition to intensive television advertising. The IEC campaign also involved CDDs, government at the state, district and village levels, and a touring bicycle team wearing “Filaria Day” t-shirts to advertise the distribution day. The enhanced campaign resulted in 88.0% treatment coverage compared to 71.0% coverage observed at study baseline (Fig 2).

While the primary aim of de Rochars’s (2005) study in Haiti was to demonstrate the effect of annual LF MDA on microfilaremia prevalence over three rounds of treatment, the study also evaluated the influence of a communication campaign on treatment coverage [23]. Despite extensive community sensitization at baseline, treatment coverage decreased during the second round of MDA. Therefore, prior to the third treatment round, researchers implemented a communication campaign consisting of radio broadcasts by community leaders and televised videos about benefits and risks of LF preventive chemotherapy. The communication campaign was associated with an increase in coverage from 53.0% to 80.8% (Fig 2).

Using a prospective cross-sectional study, Zimicki 1994 evaluated the influence of a national mass media communication campaign on full child vaccine coverage in the Philippines [24]. Prior to 1990, standard childhood vaccine distribution occurred in health centers with no accompanying IEC campaigns. In 1989, 50.7% of children were fully covered by standard vaccine campaigns. The mass media IEC campaign implemented in 1990 popularized a single day of the week as “vaccine day,” and provided information on measles vaccines in particular, as measles was a threat well known by mothers. The communication campaign resulted in 60.4% of children completing their vaccine schemes on time (Fig 2).

The pooled increase in treatment coverage from these eight studies was 21.6%, with an average absolute post-intervention coverage of 77.8% (Fig 2).

### Fixed-point vs. door-to-door delivery

In addition to intensifying IEC activities, King 2011 (described above) evaluated the influence of changing the distribution method for LF MDA [14]. Standard-of-care distribution was conducted door-to-door by health staff, achieving 49.6% treatment coverage. The following year, MDA was delivered in fixed-point community spaces including churches, schools, places of employment, shopping centers, bingo halls, and the airport. This change was informed by distributor and community feedback on the feasibility and cultural appropriateness of distribution platform. Post-intervention coverage was 71.0% (Fig 2).

Conversely, Linkins 1995 used a prospective cross-sectional study to evaluate OPV coverage in Egypt using door-to-door distribution as compared to standard-of-care fixed-point delivery. Coverage was defined as the percent of children less than five years of age who received OPV. Standard-of-care fixed-point distribution was associated with a coverage of 86.0% while door-to-door distribution was associated with 100% coverage [25] (Fig 2). Although overall program costs were higher with the more intensive doo-to-door distribution method, cost per child was comparable to fixed-point delivery given the higher coverage achieved.

### Integrated vs. vertical service distribution

Nine studies included in this review evaluated the impact on coverage of integrating distribution of community-based public health services [19, 26–33]. One such platform for integration is Child Health Days, or Child Health Weeks or Months, which are implemented routinely throughout Eastern and Southern Africa as a strategy for efficiently and comprehensively distributing packages of key preventative health services to children such as immunizations, nutritional supplementation, and insecticide-treated bed nets [38].

In Central Nigeria, Blackburn 2006 used a prospective cross-sectional study to evaluate how integration of ITN distribution with an existing LF and onchocerciasis MDA program would affect the coverage of ITN ownership [26]. Coverage was defined as the percent of children less than 5 years of age and pregnant women who owned an ITN. ITNs were distributed simultaneously with LF and onchocerciasis MDA. Prior to integrated service distribution, ITN coverage was 9.0%. ITN coverage increased to 74.0% when distributed in conjunction with the existing MDA program (Fig 2).

In 2009, a nationally integrated MDA program targeting LF, onchocerciasis, trachoma, schistosomiasis, and STH was implemented in Mali [27]. Dembele 2012 used a prospective cross-sectional study to compare program coverage achieved by national disease-specific vertical distribution in 2006 to that achieved by national integrated distribution in 2009. Program coverage was defined as the proportion of the population treated among the eligible population in targeted areas. Integrated distribution increased treatment coverage for schistosomiasis and STH from 40.1% to 71.4%, and from 40.5% to 91.5%, respectively. Onchocerciasis treatment coverage increased only slightly, from 101.6% to 103.8%. However, LF and trachoma treatment coverage decreased from 97.6% to 91.5% and 104.9% to 78.5%, respectively. Overall, treatment coverage increased 19.3% with integrated service distribution (Fig 2).

Two studies (Doherty 2010 and Oliphant 2010) used retrospective data reported in Demographic and Health Surveys (DHS) from Ethiopia, Madagascar, Tanzania, Uganda, Zambia, and Zimbabwe to evaluate how coverage of vitamin A supplementation and measles immunization campaigns were influenced by integrating these services in national Child Health Days [28, 33]. Data abstracted from DHS included the number of children age 6–59 months who received vitamin A supplementation in the 6 months preceding the survey and the number of children age 12–23 months who received a measles vaccine at any time prior to the survey. Treatment coverage was calculated as the proportion of children sampled that received services. The change in coverage was calculated by subtracting the most recent coverage estimate from the DHS report preceding integration from the DHS estimate following integration. Prior to implementation of CHDs, vitamin A supplementation coverage in Ethiopia, Madagascar, and Tanzania was 55.8%, 4.2% and 13.8%, respectively. Following integration into the CHD platform, coverage in Ethiopia decreased to 45.8%, but in Madagascar and Tanzania increased to 76.2% and 45.5%, respectively. The effect of integrating measles immunization programs into the CHD structure was also mixed. Coverage in Tanzania and Zambia did not change significantly following CHD implementation, while coverage in Zimbabwe decreased from 79.1% to 65.6%. However, coverage in Ethiopia and Uganda increased from 26.6% and 56.8% to 34.9% and 68.1%, respectively. While the effect of integrated distribution on coverage in each country varied, Doherty 2010 found that average coverage increased 47.1%, with an average absolute post-intervention coverage of 55.5%, and Oliphant 2010 found that average coverage decreased 0.8% with an absolute post-intervention coverage of 50.0% (Fig 2).

In 2007, a nationwide integrated distribution campaign of measles vaccines, deworming drugs, and vitamin A supplementation targeting children less than 59 months of age was launched in Madagascar [29]. ITN distribution was included in the campaign in 59 districts. Goodson 2012 compared cross-sectional measures of measles vaccine coverage in areas that were and were not targeted for ITN distribution. Coverage was defined as the percentage of children less than 59 months who received a measles vaccine through the campaign. In areas without concurrent ITN distribution, measles vaccine coverage was 61.5% while in areas with concurrent ITN distribution coverage was 71.0% (Fig 2).

Conversely, Grabowsky 2005 used a prospective cross-sectional study in five districts in Zambia to evaluate the influence of integration of ITN delivery within national measles vaccine campaigns [30]. Coverage was defined as the percentage of children aged 9 months to 14 years who received an ITN during a one-week measles vaccine campaign. Preceding integrated distribution, ITN coverage was 22.7%. Integrated delivery with the vaccine campaign achieved ITN coverage of 80.2% (Fig 2).

The previously described study by Habib 2017 evaluated the influence of integrated distribution in Pakistan on OPV treatment coverage [19]. The control arm used routine vertical immunization services, while the experimental arm used integrated distribution of OPV and maternal and child health services preceded by IEC activities. In the control arm, where OPV was distributed vertically with no accompanying IEC, OPV treatment coverage was 75%. In the experimental arm, which integrated distribution of OPV with other services and incorporated IEC materials, OPV treatment coverage was 82% (Fig 2).

Mwingira 2016 used a prospective cross sectional study to evaluate the influence of integrating a measles and rubella vaccine campaign with MDA for onchocerciasis and LF in Tanzania on MDA treatment coverage compared to vertical distribution [31]. In addition to integrating distribution of the vaccine and MDA, planning exercises, community sensitization, media campaigns, and monitoring and evaluation for both the vaccine campaign and MDA were integrated. Coverage was defined as the percent population who received MDA. Vertical distribution of the MDA resulted in 86% treatment coverage, while integrated distribution resulted in 93% coverage (Fig 2).

As previously described, Ndyomugyenyi 2003 evaluated how integration of schistosomiasis and STH treatment with existing community-directed onchocerciasis treatment influences onchocerciasis treatment coverage [32]. STH treatment was distributed to children aged 5 to 14 years, and onchocerciasis treatment was distributed to all eligible community members. Coverage was defined as the percentage of the eligible population who received onchocerciasis treatment. Integration improved onchocerciasis treatment coverage from 77.2% to 81.3% over standard community-directed treatment (Fig 2).

The pooled change in coverage from these nine studies demonstrated a 12.7% increase in coverage, and an average absolute post-intervention coverage of 90.0% (Fig 2).

### Community-based vs. school-based delivery

Three articles evaluated changes in MDA treatment coverage when delivered as community-based campaigns as opposed to standard school-based programs [7, 32, 34]. School-based programs are characterized by distribution of health services to school-aged children by teachers and/or health staff in schools. Children who are not enrolled in schools are typically invited to attend treatment days to receive the health service, and adults are not targeted for treatment. In these studies, community-based programs combined school-based distribution to target children enrolled in school as well as distribution in community spaces to target unenrolled children and adults. In both studies treatment coverage was calculated as the proportion of targeted individuals who received treatment.

The previously described study in Tanzania (Massa 2009) compared coverage of standard-of-care school-based MDA to community-based MDA for schistosomiasis in enrolled and unenrolled school-age children [7]. Two rounds of community-based delivery were implemented and coverage was estimated. Among enrolled school-aged children, the first round of the intervention achieved similar coverage to the standard-of-care (80.3% compared to 82.1%, respectively). Among unenrolled children, however, the intervention achieved higher coverage than the standard-of-care (80.0% compared to 59.2%, respectively). Similar results were observed following a second round of the intervention; coverage of children enrolled in school was not significantly different from the standard-of-care among enrolled children, whereas coverage amongst unenrolled children increased relative to the standard-of-care among unenrolled children (80.7% in intervention cluster compared to 78.4% in standard-of-care cluster) (Fig 2).

In Arua District, Uganda, Ndyomugyenyi 2003 used a community-RCT to compare distribution of schistosomiasis and STH treatment through community-based distribution and school-based distribution [32]. Coverage was defined as the percentage of children aged 5 to 14 years who received treatment. School-based distribution by teachers in primary school was associated with coverage of 79.0%, while community-based delivery by community drug distributors was associated with treatment coverage of 85.0% (Fig 2).

Another study (Oshish 2011) used prospective repeat cross-sectional measures to evaluate changes in schistosomiasis MDA treatment coverage of school-age children (6–18 years) and adults when transitioning from standard-of-care school-based MDA to community-based MDA [34]. Standard-of-care delivery had 94.0% coverage of enrolled school-aged children, 68.0% coverage of unenrolled school-aged children, and did not target adults. Community-based delivery via schools, health centers, markets, mosques, and community leaders’ residences achieved 97.9% coverage of enrolled children, 90.0% coverage of unenrolled children, and 73.9% coverage of adults, with an overall coverage of 82.5% (Fig 2).

The pooled change in coverage from these three studies demonstrated an 11.8% increase in coverage, and an average absolute post-intervention coverage of 94.5% among school-aged children (Fig 2).

### Distributions managed by NGOs vs. MOH

One study (Ladner 2014) evaluated the influence of campaign management source on the treatment coverage in community-based public health programs [35]. The study used linear regression to retrospectively evaluate factors that were associated with Gardasil vaccine coverage among girls age 9 to 13 years in several low- and middle-income countries using data from the Gardasil Access Program (GAP). Coverage was calculated as the proportion of targeted girls who received the full vaccine course. Distribution management by an NGO compared to an MOH was found to be significantly associated with higher vaccine coverage (p = 0.05). Across 14 countries, programs under NGO management achieved 93.1% coverage, while programs under MOH management achieved 87.3% coverage, not accounting for distribution duration or community involvement (Fig 2).

## Discussion

Optimizing treatment coverage in community-based public health programs has the potential to dramatically improve health and development outcomes targeted by these programs, particularly among marginalized groups [39–41]. This systematic review of the literature identified nine specific strategies that were used to increase treatment coverage in community-based health programs. Strategies that increased community participation in and ownership of distribution activities, including community-directed distribution, incentives to increase distributor motivation, and distribution along kinship networks, demonstrated the largest positive influence on treatment coverage. Strategies that increased access to services by bringing them to the community, including door-to-door distribution and community-based delivery, demonstrated the highest absolute post-intervention coverage.

Community-directed treatment distribution demonstrated the largest impact on coverage of any intervention in this review. This strategy proved particularly successful at distributing services in a hard-to-reach nomadic population that otherwise had no access to health services, and in an urban population, where achieving high MDA coverage is challenging [4, 11]. However, amongst the included studies, community-directed treatment strategies rarely achieved absolute coverage levels of 80% or higher. Enhancing community-directed treatment by encouraging distribution on a smaller, kinship scale both increased coverage and achieved high absolute coverage [15].

Integrated delivery of community-based public health services demonstrated a high absolute post-intervention coverage. Programs and governments are increasingly integrating service distribution to streamline delivery of a variety of services and reduce costs [42]. However, as demonstrated in this review, there is significant heterogeneity in the impact of integration by setting and service. This study found that integration of novel ITN distribution programs with existing drug distribution programs successfully increased coverage of ITN ownership, but that integration of existing vertical MDA programs with other existing MDA programs resulted in only a minor increase in coverage of either program. This suggests that integration of existing services requires more than simple co-delivery, and should be designed just as carefully as programs that pursue integration of novel services. More research is needed to establish how integration may affect cost-effectiveness of distribution.

Evidence from multiple settings indicates that individual participation in community-based public health programs is limited by lack of information or by the provision of misinformation regarding side effects, benefits of treatment, and distribution activity schedules [18, 22]. Providing information regarding disease transmission factors and the schedule of distribution activities can increase the probability of an individual’s participation up to five-fold by stimulating demand for a program [18, 43]. In this review, enhanced IEC activities had a strong positive influence on treatment coverage by providing the information necessary to encourage and support participation. Evidence suggests that addressing community specific information gaps within IEC activities and repeating these activities prior to each round of distribution may offer additional benefit [23]. Because IEC activities are regularly included as a core component of public health programs, increasing the breadth of IEC activities is likely to be a feasible and low-cost strategy to increase treatment coverage.

NGO program management resulted in one of the highest absolute coverage estimates of the strategies evaluated in this review, although the evidence is based on data from a single study. NGOs may face fewer bureaucratic challenges than governments, facilitating quicker implementation of vertical programs [35]. This reflects a common tension that exists in some resource limited countries; NGOs may have greater flexibility than country governments in designing and delivering health programs, but may not offer the sustainability of local leadership. For example, in some settings, NGOs may be able to more easily provide distributor incentives such as strong supervision, training, and financial incentives [44]. These distributor incentives demonstrated positive influences on treatment coverage in this review [16].

There are a number of strengths to this analysis. The primary aim of all but one of the included studies was to evaluate the impact of strategies on MDA treatment coverage. All studies employed rigorous study designs, included large sample sizes and were published in peer-reviewed journals. Strategies that were evaluated in more than one study and location demonstrated similar effects on coverage, indicating that the impact of these strategies may be consistent across settings and distribution channels. While we were concerned that inclusion of articles that reported the first round of MDA in an area would artificially inflate our change in coverage estimate, exclusion of those studies did not change the overall estimate, and were therefore kept in this review [11, 26, 30]. As coverage does not necessarily increase with repeat distribution in a given area, observed improvements in coverage were likely to be associated with implementation of the strategies, rather than observed as a temporal consequence of the program [7, 23]. This is also the only quantitative review of strategies to increase treatment coverage of public health services known to the authors.

However, there are also several limitations to this analysis. Included studies utilized self-reported coverage estimates, which may be subject to recall bias [45]. In addition, where studies employed more than one strategy to increase coverage, the magnitude of change may be over-estimated as it was not possible to parse out the strategy-specific effect in a single population. Finally, we lacked sufficient information on cluster-level sample sizes so we assumed independence between individuals in clusters. Therefore, the 95% CIs calculated for the change in coverage for each intervention does not account for clustering present in some of the studies.

### Conclusion

Delivering community-based public health interventions at scale can be challenging and impact is often limited by the ability to reach targeted populations. Identifying strategies that can improve treatment coverage is relevant for a number of ongoing and planned interventions. In this review, we identify several strategies that appear to demonstrate a positive impact on the treatment coverage in community-based public health programs. Strategies that increased the community’s role in and ownership of distribution events had the largest positive influence on change in treatment coverage. In contrast, strategies that achieved the highest absolute coverage did so by increasing access to services through door-to-door and community-based distribution. In areas with insufficient coverage, we suggest that public health officials and implementation partners consider the ‘supply’ and ‘demand’ side factors influencing community member participation in programs. For example, if programs are not reaching targeted populations with services than they may consider transitions in delivery strategy (ex. door-to-door delivery vs. fixed point delivery) in order to increase coverage. On the other hand, if coverage is low because community members choose not to comply, then programs may wish to consider strategies such as community-directed treatment or intensified IEC campaigns. This study provides a menu of potential strategies from which public health officials and partners can evaluate which coverage interventions may be most appropriate within their own setting. Additional analyses to determine the cost-effectiveness and feasibility of each strategy, or combinations of strategies, are needed to inform program and policy decisions to optimize these programs at scale. These findings are particularly important for studies that aim to interrupt the transmission of disease by providing preventative interventions with extremely high coverage.

## Supporting information

S1 Checklist PRISMA checklist. (DOC)

S1 Table Percent change in coverage achieved by each strategy. Pooled and individual targeted and treated population sizes and corresponding treatment coverage estimates achieved by each strategy compared to standard-of-care distribution activities.
(DOCX)
