# Supplementary material for: Strategies to improve treatment coverage in community-based public health programs: A systematic review of the literature
Source: PLoS Negl Trop Dis. 2018 Feb 8;12(2):e0006211. doi: 10.1371/journal.pntd.0006211 (PMC5805161; doi:10.1371/journal.pntd.0006211)
Supplement: S1 Table — Pooled and individual targeted and treated population sizes and corresponding treatment coverage estimates achieved by each strategy compared to standard-of-care distribution activities. (DOCX) [file pntd.0006211.s002.docx]

| **S1 Table.** Percent change in coverage achieved by each strategy. Pooled and individual targeted and treated population sizes and corresponding treatment coverage estimates achieved by each strategy compared to standard-of-care distribution activities. | | | | | | | | | | | | | | |
| --- | --- | --- | --- | --- | --- | --- | --- | --- | --- | --- | --- | --- | --- | --- |
|  |  |  | **Intervention** | | | | | ^§^**Control** | | | | | **Change** | |
| **Strategy** | **Author** | **Country** | **Targeted (N)** | **Treated (N)** | **Coverage** | **(95% CI)** | **Year** | **Targeted (N)** | **Treated (N)** | **Coverage** | **(95% CI)** | **Year** | **% Change** | **(95% CI)** |
| **Community-Directed Treatment** |  |  | **23,640** | **18,693** | **79.1%** | **(78.5, 79.6)** | **--** | **16,023** | **8,477** | **52.9%** | **(52.1, 53.7)** | **--** | **26.2%** | **(25.2, 27.1)** |
|  | Akogun 2012 | Nigeria | 483 | 322 | 66.7% | (62.2, 70.8) | 2006-2009 | 218 | 0 | 0% | (0.0, 2.2) | 2006-2009 | 66.7% | (62.5, 70.9) |
|  | Babu 2006 | India | 4,854 | 4,451 | 91.7% | (90.9, 92.5) | 2004 | 1,221 | 838 | 68.6% | (65.9, 71.2) | 2004 | 23.1% | (20.4, 25.8) |
|  | Gyapong 2000 | Ghana | 1,494 | 1,103 | 73.8% | (71.5, 76.0) | -- | 1,410 | 694 | 49.2% | (46.6, 51.9) | -- | 24.6% | (21.2, 28.0) |
|  | Halwindi 2010 | Zambia | 5,922 | 3,868 | 65.3% | (64.1, 66.5) | 2006-2007 | 6,518 | 2,144 | 32.9% | (31.8, 34.1) | 2006-2007 | 32.4% | (30.7, 34.1) |
|  | Massa 2009 | Tanzania | 9,140 | 7,412 | 81.1% | (80.3, 81.9) | -- | 4,938 | 4,002 | 81.0% | (79.9, 82.1) | -- | 0.0% | (-1.3, 1.4) |
|  | Wamae 2006 | Kenya | 1,746 | 1,537 | 88.0% | (86.4, 89.5) | 1997-1999 | 1,718 | 799 | 46.5% | (44.1, 48.9) | 1997-1999 | 41.5% | (38.7, 44.3) |
| **Kinship-Enhanced** | **Katabarwa 2010** | **Uganda** | **894** | **837** | **93.7%** | **(91.8, 95.1)** | **2005-2006** | **1,500** | **1,038** | **69.3%** | **(66.8, 71.5)** | **2005-2006** | **24.5%** | **(21.7-27.3)** |
| **Incentives** |  |  | **1,120** | **592** | **52.9%** | **(49.4, 55.8)** | **--** | **1,120** | **309** | **27.6%** | **(25.0, 30.3)** | **--** | **25.3%** | **(21.3, 29.1)** |
|  | Calderon-Ortiz 1996 | Mexico | 100 | 93 | 93.5% | (85.6, 96.9) | 1994 | 100 | 21 | 21.1% | (13.8, 30.5) | 1994 | 72.4% | (63.1, 81.7) |
|  | Muhumuza 2013 | Uganda | 1,020 | 499 | 48.9% | (44.4, 53.4) | 2012 | 1,020 | 288 | 28.2% | (22.9, 33.6) | 2011 | 20.7% | (16.6, 24.8) |
| **IEC** |  |  | **292,108** | **227,116** | **77.8%** | **(77.6, 77.9)** | **--** | **240,362** | **134,911** | **56.1%** | **(55.9, 56.3)** | **--** | **21.6%** | **(21.4, 21.8)** |
|  | Cantey 2010 | India | 1,770 | 1,053 | 59.5% | (57.2, 61.8) | 2007 | 1,679 | 876 | 52.2% | (49.8, 54.6) | 2007 | 7.3% | (4.0, 10.6) |
|  | Habib 2017 | Pakistan | 29,126 | 24,465 | 84.0% | (83.6, 84.4) | 2013-2014 | 28,760 | 21,570 | 75.0% | (74.5, 75.5) | 2013-2014 | 9.0% | (8.3, 9.7) |
|  | King 2011 | American Samoa | 42,836 | 30,414 | 71.0% | (70.6, 71.4) | 2003 | 42,836 | 21,247 | 49.6% | (49.1, 50.1) | 2002 | 21.4% | (20.8, 22.0) |
|  | Njomo 2014 | Kenya | 62,455 | 45,087 | 72.2% | (71.8, 72.5) | 2012 | 10,902 | 7,675 | 70.4% | (69.5, 71.3) | 2012 | 1.8% | (0.9, 2.7) |
|  | Rahman 2012 | Iraq | 1,155 | 1,012 | 87.6% | (85.5, 89.4) | 2007 | 1,115 | 412 | 36.9% | (34.1, 39.9) | 2006 | 50.6% | (47.2, 54.0) |
|  | Ramaiah 2006 | India | 3,862 | 3,399 | 88.05 | (86.9, 89.0) | 2002 | 4,182 | 2,969 | 71.0% | (69.6, 72.4) | 2001 | 17.0% | (15.3, 18.7) |
|  | de Rochars 2005 | Haiti | 150,000 | 121,139 | 80.8% | (80.6, 81.0) | 2002 | 150,000 | 79,713 | 53.0% | (52.9, 53.4) | 2001 | 27.8% | (27.5, 28.1) |
|  | Zimicki 1994 | Philippines | 903 | 545 | 60.4% | (57.1, 63.5) | 1990 | 887 | 449 | 50.7% | (47.3, 54.0) | 1989 | 9.7% | (5.2, 14.4) |
| **Fixed-Point Delivery** | **King 2011** | **American Samoa** | **42,836*** | **30,414** | **71.0%** | **(70.6, 71.4)** | **2003** | **42,836*** | **21,247** | **49.6%** | **(49.1, 50.1)** | **2002** | **21.4%** | **(20.8, 22.0)** |
| **Door-to-Door Delivery** | **Linkins 1995** | **Egypt** | **7,003** | **7,003** | **100%** | **(99.0, 100)** | **1993** | **7,003** | **6,026** | **86.0%** | **(85.2, 86.8)** | **1992** | **14.0%** | **(31.1, 14.8)** |
| **Integrated Delivery** |  |  | **57,239,806** | **51,515,695** | **90.0%** | **(89.9, 90.0)** | **--** | **40,132,663** | **31,029,345** | **77.3%** | **(77.3, 77.3)** | **--** | **12.7%** | **(12.6, 12.7)** |
|  | Blackburn 2006 | Nigeria | 2,828 | 2,092 | 74.0% | (67.0, 82.0) | 2004 | 2,828 | 254 | 9.0% | (5.0, 12.0) | 2003 | 65.0% | (63.1, 66.9) |
|  | Dembele 2012 | Mali | 34,985,573 | 30,807,173 | 88.1% | (88.0, 88.1) | 2009 | 19,904,856 | 13,685,016 | 68.8% | (68.7, 68.8) | 2006 | 19.3% | (19.2, 19.3) |
|  | Doherty 2010 | Several | 24,081 | 13,375 | 55.5% | (54.9, 56.2) | -- | 57,451 | 4,864 | 8.5% | (8.2, 8.7) | -- | 47.1% | (46.4, 47.7) |
|  | Goodson 2012 | Madagascar | 1,629 | 1,156 | 71.0% | (65.7, 75.8) | 2006 | 291 | 178 | 61.5% | (53.6, 68.9) | 2006 | 9.5% | (3.5, 15.5) |
|  | Grabowsky 2005 | Zambia | 2,074 | 1,663 | 80.2% | (78.4, 81.9) | 2003 | 2,074 | 471 | 22.7% | (20.9, 24.6) | 2002 | 57.5% | (55.0, 60.0) |
|  | Habib 2017 | Pakistan | 29,126 | 24,465 | 84.0% | (83.6, 84.4) | 2013-2014 | 28,760 | 21,570 | 75.0% | (74.5, 75.5) | 2013-2014 | 8.5% | (6.8, 10.1) |
|  | Mwingira 2016 | Tanzania | 22,161,109 | 20,648,355 | 93.0% | (92.9, 93.1) | 2014 | 20,101,582 | 17,298,697 | 86.0% | (86.0, 86.1) | 2013 | 7.0% | (6.9, 7.0) |
|  | Ndyomugyenyi 2003 | Uganda | 2,500 | 2,032 | 81.3% | (79.7, 82.8) | -- | 2,530 | 1,954 | 77.2% | (75.5, 78.8) | -- | 4.1% | (1.9, 6.3) |
|  | Oliphant 2010 | Several | 30,886 | 15,382 | 49.8% | (49.2, 50.4) | -- | 32,291 | 16,340 | 50.6% | (50.1, 51.1) | -- | -0.8% | (-1.6, 0.0) |
| **Community-Based Delivery**** |  |  | **352,966** | **333,429** | **94.5%** | **(94.4, 94.5)** | **--** | **372,105** | **307,391** | **82.6%** | **(82.5, 82.7)** | **--** | **11.9%** | **(11.7, 12.0)** |
|  | Massa 2009 | Tanzania | 9,140 | 7,412 | 81.1% | (80.3, 81.9) | -- | 4,938 | 4,002 | 81.0% | (79.9, 82.1) | -- | 0.0% | (-1.3, 1.4) |
|  | Ndyomugyenyi 2003 | Uganda | 590 | 502 | 85.0% | (81.9, 87.8) | -- | 359 | 284 | 79.0% | (74.5, 83.1) | -- | 6.0% | (0.9, 11.1) |
|  | Oshish 2011^¶^ | Yemen | 343,236 | 325,515 | 94.8% | (94.7, 94.9) | 2009 | 366,808 | 303,105 | 82.6% | (82.5, 82.8) | 2008 | 12.2% | (12.1, 12.3) |
| **NGO Management** | **Ladner 2014** | **Several** | **59,640** | **55,507** | **93.1%** | **(92.9, 93.3)** | **--** | **185,916** | **162,279** | **87.3%** | **(87.1, 87.4)** | **--** | **5.8%** | **(5.5, 6.1)** |
| ^§^ Generally standard-of-care delivery method | | | | | | | | | | | | | | |
| IEC: Information, Education, Communication Activities; NGO: Non-Governmental Organization | | | | | | | | | | | | | | |
| * Targeted population estimate from 2004 | | | | | | | | | | | | | | |
| ** Includes only school-aged children | | | | | | | | | | | | | | |
| ^¶^ Does not include coverage among adults | | | | | | | | | | | | | | |
